# Supplementary material for: Complexome profiling of the Chlamydomonas psb28 mutant reveals TEF5 as an early PSII assembly factor
Source: Plant Cell. 2025 Mar 17;37(6):koaf055. doi: 10.1093/plcell/koaf055 (PMC12164590; doi:10.1093/plcell/koaf055)
Supplement: koaf055_Supplementary_Data [file koaf055_supplementary_data.zip › psb28_interactive.zip!/plots/intensity/qualityPlots/peptides/ATPC_Cre06.g259900.t1.2.html]

### Cre06.g259900.t1.2

Trivial name: ATPC  
SeqCoverage [median]: 55.90  
Mass: 38.76 kDa  
  
 

| Sequence | MissedCleavage | Mass [Da] | wtIntensity | muIntensity | Score |
| --- | --- | --- | --- | --- | --- |
| AQEAVVNGR | 0 | 942.49 | 4736.77 | 5959.84 | 121.05 |
| AQEAVVNGRPFSENLVK | 1 | 1856.97 | 76366.18 | 140058.69 | 137.17 |
| ELKKGLTVQYNK | 2 | 1419.81 | 0.00 | 1230.65 | 134.56 |
| ELTAMGVK | 0 | 847.45 | 168997.03 | 72744.11 | 117.16 |
| FISLINSNPTIQTLLPMTPMGELCDVDGKCVDAADDEIFKLTTK | 2 | 4796.37 | 6790.57 | 0.00 | 45.46 |
| GAQYFAR | 0 | 811.40 | 233501.72 | 169440.22 | 149.06 |
| GGEFAVER | 0 | 863.41 | 620344.04 | 518183.31 | 159.46 |
| GGEFAVEREK | 1 | 1120.55 | 51610.46 | 63902.57 | 107.06 |
| GLTVQYNK | 0 | 921.49 | 725785.55 | 573845.10 | 226.78 |
| ITDAMKLVAAAK | 1 | 1230.70 | 22199.10 | 42788.95 | 98.35 |
| ITQELAEIVGGAAATSG | 0 | 1586.82 | 644104.49 | 552813.79 | 306.06 |
| KGAQYFAR | 1 | 939.49 | 113521.55 | 86280.95 | 257.98 |
| KGLTVQYNK | 1 | 1049.59 | 85267.31 | 75568.00 | 241.96 |
| KQYNIVK | 1 | 891.52 | 18656.95 | 41537.04 | 158.53 |
| MNAMNNASDNAK | 0 | 1279.53 | 26047.45 | 45734.18 | 256.38 |
| NTQKITDAMK | 1 | 1148.59 | 1496.89 | 1868.43 | 151.83 |
| NTQKITDAMKLVAAAK | 2 | 1701.94 | 0.00 | 1583.97 | 69.40 |
| PFSENLVK | 0 | 932.50 | 32456.40 | 20099.25 | 166.68 |
| QYNIVK | 0 | 763.42 | 119152.64 | 21857.44 | 174.60 |
| RAQEAVVNGR | 1 | 1098.59 | 603.50 | 1863.17 | 75.51 |
| RAQEAVVNGRPFSENLVK | 2 | 2013.08 | 32481.34 | 36222.66 | 104.88 |
| RKQYNIVK | 2 | 1047.62 | 48105.73 | 49731.38 | 159.20 |
| SFSLGAAPSTK | 0 | 1064.55 | 648256.14 | 587519.66 | 203.97 |
| SLQEALASELAAR | 0 | 1357.72 | 766155.19 | 751603.10 | 290.61 |
| SVLLVVLTGDR | 0 | 1170.70 | 217367.68 | 315498.94 | 242.83 |
| VLYGVNQR | 0 | 947.52 | 642316.64 | 728048.71 | 207.01 |
| YRELTAMGVK | 1 | 1166.61 | 331322.20 | 252301.79 | 154.05 |
|  |  |  | 5637643.5 | 5158285.9 |  |
